# Arbuscular mycorrhizal fungi and their response to pesticides

**DOI:** 10.1002/ps.5220

**Published:** 2018-10-29

**Authors:** Karin Hage‐Ahmed, Kathrin Rosner, Siegrid Steinkellner

**Affiliations:** ^1^ Division of Plant Protection, Department of Crop Sciences University of Natural Resources and Life Sciences Vienna Tulln Austria

**Keywords:** arbuscular mycorrhizal fungi, pesticides, fungicides, insecticides, herbicides, nontarget effect

## Abstract

Arbuscular mycorrhizal fungi (AMF) form symbioses with the majority of plant species and can provide multiple benefits to the host plant. In agro‐ecosystems, the abundance and community structure of AMF are affected by agricultural management practices. This review describes and discusses current knowledge on the effects of inorganic and organic chemical pesticides on AMF in the conflicting area between agricultural use and environmental concerns. Variable effects have been reported following chemical pesticide use, ranging from neutral to positive and negative. Moreover, a species‐specific reaction has been documented. The reported effects of pesticides on arbuscular mycorrhizal symbiosis are very diverse, and even when the same substance is investigated, the results are often contradictory. These effects depend on many parameters, such as the active substance, the mode of action, the mode of application and the dosage. In the field, determinants such as the physico‐chemical behavior of the active substances, the soil type and other soil microorganisms contribute to the fate of pesticides and thus the amount of active substances to which AMF are exposed. This review highlights that the fate of AMF following pesticide use needs to be addressed in a broader agro‐ecosystem context. © 2018 The Authors. *Pest Management Science* published by John Wiley & Sons Ltd on behalf of Society of Chemical Industry.

## 1 INTRODUCTION

Arbuscular mycorrhizal fungi (AMF) form symbioses with the majority of plant species and can provide multiple benefits to the host plant, such as increased nutrient uptake, drought resistance and resistance to pathogens.^1^ Mycorrhizal symbioses in agro‐ecosystems are affected by a broad range of farming practices, such as soil tillage, fertilization and plant protection measures.^2^, ^3^, ^4^ In integrated pest management (IPM), all available agronomical and plant protection tools should be considered and combined to secure food production and to reduce the development of populations of harmful organisms in an ecologically and economically sound manner. This approach includes cultivar selection, fertilization balance, adequate cultivation techniques and sustainable biological, physical and nonchemical methods. The use of reduced‐risk pesticides should ensure minimal side effects on human health, nontarget organisms and the environment.

**Figure 1:**
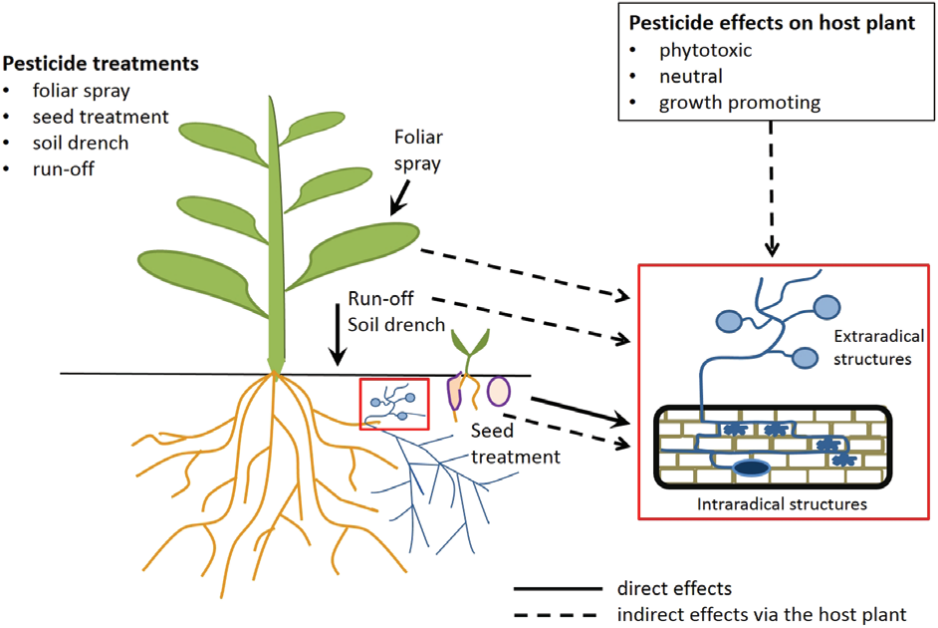
Putative ways of exposure of AMF to pesticides.

In the field, AMF structures such as spores and hyphae can be exposed to active substances when these substances are either applied as soil drench, seed treatment or foliar spray, or are present in run‐off from leaf application or drift to the soil. Additionally, indirect effects on arbuscular mycorrhizal (AM) symbiosis mediated by changes in the host plant physiology can occur (Fig. 1). To combine the benefits from AMF and chemical plant protection measures, putative nontarget effects on AMF need to be assessed. Active pesticide substances have different modes of action that determine the principal hazard potential. Consequently, a key goal is to identify harmful substances and then assess the risk of exposure to such substances during the lifecycle of AMF within a crop scenario. Furthermore, understanding what strategies AMF have evolved to tolerate critical doses of harmful substances and which management practices can support AMF recovery is necessary. This review describes and discusses current knowledge on the effects of pesticides on AMF in the conflicting area between agricultural use and environmental concerns. We focus on inorganic and organic chemicals currently approved in the European Union to reflect an IPM‐directed use of pesticides (Table S1). Chemicals that have been phased out and microbial biopesticides are not included.

## 2 AMF DEVELOPMENT AND IMPLICATIONS FOR TEST SYSTEMS/PESTICIDE TESTING

AMF are obligate biotrophic fungi with a very intimate relationship with their host plant. This intimate relationship is characterized by bidirectional nutrient transfer between the plant and the fungal partner and involves specialized fungal structures (e.g., arbuscules) within the root cortex cells.^1^ Certain stages in AM development are necessary to establish AM symbiosis and to guarantee fungal continuity in the field. Those stages include the pre‐symbiotic phase (i.e., spore germination, germ tube elongation, hyphal branching and host contact), active symbiosis within the roots and its extraradical mycelium in the surrounding soil, and the establishment of vesicles and spores that survive periods without an available host plant. Interferences at certain stages of development can have deleterious effects on AM establishment and fungal continuity in the field. Thus, negative effects of pesticides in such key stages should be minimized.

Integrating these different aspects of AMF lifecycle into test systems and experimental designs is a great challenge. Moreover, pesticide testing requires specific considerations as well. Important considerations for experimental designs include the type of pesticide, the active compounds and their respective product formulations and the application mode, such as foliar spray, soil and seed treatment. Furthermore, the particular challenge lies within distinguishing between direct and indirect effects (e.g., *via* changes in the host plant physiology) on AMF.

In the pre‐symbiotic phase, the direct effects of active substances and their respective formulations on spore germination and germ tube development can be assessed using *in vitro* experiments (supporting information Table S2). AMF spores can germinate and form a pre‐symbiotic mycelium in axenic culture, but they cannot grow continuously without a host plant. In AM‐specific *in vitro* systems, Ri T‐DNA‐transformed roots^5^ or whole autotrophic plants^6^ are cultured on artificial growth medium and are associated with an AMF strain. A physical separation of root compartments and hyphal compartments allows the study of the effects of substances on extraradical mycelia and sporulation without direct contact of the substance with the host. Furthermore, dose–response curves can be generated, and the effects of pure active substances and their respective product formulations can be compared. These approaches also allow us to study the effects on active symbiosis and the extraradical mycelium and to discern between direct and indirect effects. However, Ri T‐DNA‐transformed roots lack photosynthetic tissue, a normal hormone balance and physiological source–sink relationships compared with an autotrophic plant.^7^ Thus, *in vitro* systems using autotrophic plants could provide more realistic information on the effects of active substances on AMF colonization and AM symbiosis.^6^, ^8^ Another limitation is the limited availability of different AMF taxa *in vitro* due to the laborious establishment and time‐consuming maintenance of such cultures. Moreover, not all AMF species are able to develop normally under *in vitro* conditions, and their behavior under natural soil conditions might differ.

Pot and field experiments investigate host plants and their symbionts under natural or controlled conditions in soil and substrates, respectively (Table S1 (Appendix S1)). In such settings, single AMF strains or whole AMF communities can be studied with different host plant species and genotypes. Once applied to the plant or soil, the persistence of pesticides and, more importantly, the bioavailability of these compounds or their metabolites within the different soils defines how long AMF units can be exposed to putative harmful substances. As recently reviewed, this persistence and the bioavailability depend greatly on environmental factors, physico‐chemical properties of the substance, soil type, soil pH, moisture, organic matter and the ability of the local soil microflora to degrade the respective substance.^9^, ^10^ The fate of pesticides in the soil is beyond the scope of this review, but we would like to point out that variations in pot and field experiments can be caused, apart from other things, by differences in soil parameters.^11^, ^12^

A special type of pot experiment is the use of rhizoboxes with separated root and hyphal compartments that allow study of the direct and indirect effects of pesticides on AMF. However, one limitation of pot experiments in general is the difficult establishment of a root‐colonizing AMF community that reflects the diversity in the field.^12^ In field experiments, AMF communities can be studied in their natural habitat, and other factors, such as crop rotations and land management practices, can be included in the assessment. Even long‐term studies are possible. A particular challenge is the testing of herbicide effects on AMF in pot and field experiments. As obligate biotrophic fungi, AMF react to die‐off in their host plants for self‐evident reasons. Therefore, a clear impact on AMF is observed when a herbicide is applied directly to a host plant. Selective herbicide effects have been evaluated most frequently for the remaining crop plant. For nonselective herbicides such as glyphosate, two scenarios are plausible: the use of herbicide‐tolerant crop plants or evaluation in the subsequent crop. For the latter, a suitable control could be mechanical weed control by hoeing, steaming or flaming. As noted below, tillage systems are known to affect AMF by itself; therefore, they are not suitable for use as conclusive control treatments. In any case, the comparison of dying plants due to herbicide treatment and untreated growing control plants is inappropriate for drawing conclusions on herbicidal side effects.

In conclusion, each system has its advantages and limitations, but most importantly, comparisons between these different systems are difficult, in part due to incomplete or insufficient data on the test compound itself (Table S1).

## 3 EXPOSURE OF AMF TO PESTICIDES DURING CROP GROWTH

### 3.1 Persistence of AMF

The persistence of AMF in the field depends on the formation and survival of fungal structures inside and outside plant roots. In general, AMF spores and colonized root pieces are considered the most relevant survival structures, even in the absence of a viable host plant. By contrast, hyphae survive in association with a viable host plant. Even under natural conditions, spore density and hyphal density are subject to seasonal variations.^13^ Moreover, the AMF species and their individual colonization strategies determine which fungal structures (i.e., spores, colonized root pieces or hyphal fragments) are relevant for survival and the establishment of a new symbiotic relationship after the absence of a host plant.^14^

In agro‐ecosystems, the land use type, the farming system, the tillage system and the fertilization strategy are major influencing factors on AMF persistence and development. Local AMF communities are periodically challenged by fast host‐plant turnover, crop rotations and soil management, especially in annual crops. There is evidence that tillage systems, which are used to turn the soil, can negatively affect AMF by destroying the extraradical hyphal network; by contrast, no‐till systems can foster AMF and result in increased benefits for the host plant due to better plant phosphorus uptake and soil aggregate stability.^2^, ^3^, ^4^ Therefore, the persistence of AMF following pesticide use needs to be considered in a broader context. The inclusion of agricultural practices that secure a reservoir of AM fungal units and provide a habitat that supports their recovery after exposure and inactivity is essential. From that point of view, little soil disturbance provides good inoculum by leaving the established mycelial network rather undisturbed. Of course, pesticide application can be an additional challenge in the key stages of AMF development. In the field, pesticide‐affected AMF persistence also depends on pesticide treatments in the preceding crop and on a potential post‐harvest or pre‐seeding herbicide treatment. In any case, it will be co‐determined by the transport and fate of pesticides in different soils. To date, the immediate effect on the persistence of AMF has hardly been explored, and a stronger focus has been placed on the development of AMF.

### 3.2 Direct effects on AMF in the pre‐symbiotic phase

Vital AMF spores and vesicles in root pieces are the key fungal propagules for initiating AMF symbiosis in young seedlings in disturbed soil. In this pre‐symbiotic phase, newly germinated AMF hyphae have very limited time to find a suitable host.^1^, ^15^ Without successful establishment of a symbiotic relationship within this time frame, the fungal units die off. In this sensitive establishment phase, interference caused by plant protection measures might impact the development of the whole symbiosis. Consequently, direct negative effects of plant protection products on spore germination and mycelial growth in particular should be avoided during this sensitive phase.

To date, the mechanisms by which pesticides interact with AMF are poorly understood. Based on *in vitro* studies, there is evidence that fungicides such as flutolanil, azoxystrobin, fenpropimorph and fenhexamid inhibit spore germination of *Rhizophagus irregularis* in a fungistatic and nonfungicidal manner (i.e., after the transfer to fungicide‐free medium, spores can germinate) (Table S2).^8^, ^16^ Under field conditions, AMF spores can be exposed to inhibiting active substances for a certain period of time. To overcome this threat, AMF spores remain in an ungerminated state. Subsequently, when the active substance is immobilized, reduced, metabolized or even degraded, AMF spores can germinate and associate with crop plants. Such direct inhibitory effects are rarely documented in the available literature (Table S2). However, the results from soil substrate and gelled growth medium experiments differ. For the latter, negative effects of fenhexamid and fenpropimorph on germ tube length were found at concentrations of 9.3 mg L^−1^ (IC_50_) and 6.4 mg L^−1^ (IC_50_), respectively (Table S2).^16^ Both substances are registered as foliar fungicides; thereby following good agricultural practices, a direct impact on AMF in the field is very unlikely, as those levels would not get to the soil. In general, fungicides are mostly applied as foliar sprays (with very limited basipetal transport) or as seed treatments. By contrast, many herbicides are applied pre‐seeding or pre‐emergence and are therefore directly on/into the soil. Therefore, the risk potential of herbicides directly contacting AMF in soil might be higher, even under good agricultural practices.

Although there has been considerable interest in AMF–herbicide interactions (Table S1), information on the direct mechanisms behind AMF–herbicide interactions is limited. According to *in vitro* studies (Table S2), all tested herbicides showed a neutral or even positive effect on AMF at concentrations up to the recommended field rate, even though very few studies are available on the direct effects of herbicides on AMF. The synthetic auxin picloram, which can be absorbed *via* foliage or roots, did not affect the hyphal biomass or hyphal viability of *R. irregularis*. The results from this *in vitro* experiment are supported by field data, in which AMF colonization was reduced only when picloram resulted in changes in host quality and quantity.^17^ For the leaf and soil active herbicide chlorotoluron (photosynthesis inhibitor) or for soil active mixtures of bifenox (protoporphyrinogen oxidase inhibitor) and mecoprop (synthetic auxin) or of mecoprop, ioxynil (photosynthesis inhibitor) and clopyralid (synthetic auxin), only a trend is available based on laboratory data, which could not be confirmed clearly *via* accompanying field experiments.^18^ In the absence of a host plant, herbicides containing chlorotoluron seem to have no effect on AMF spore germination, whereas herbicides containing bifenox and mecoprop or mecoprop, ioxynil and clopyralid inhibited spore germination at lower concentrations but were stimulatory at higher concentrations.

Currently, the most discussed leaf‐absorbed herbicide glyphosate (an inhibitor of enolpyruvylshikimate‐3‐phosphate synthase) does not affect AMF spore germination and hyphal growth when used at concentrations up to the recommended field rate.^19^, ^20^ Based on an *in vitro* study, the direct inhibition of AMF due to glyphosate occurred only when glyphosate was applied above the recommended application rate. Moreover, a species‐specific reaction has been documented. The inhibitory effect of glyphosate concentrations above the recommended field rate was more pronounced for the AMF species *Claroideoglomus etunicatum* than for *Scutellospora heterogama* and *Gigaspora margarita*.^19^

For glyphosate, the pesticide concentration that causes 50% inhibition (IC_50_) has been determined for both the extraradical mycelium growth and mycelial sporulation of mycorrhizal fungi.^21^ The lowest values calculated for glyphosate were 0.5 ppm for mycelial growth and 0.4 ppm for sporulation; for the glyphosate metabolite aminomethylphosphonic acid (AMPA), the IC_50_ values increased by a factor of > 7 and > 12, respectively. Therefore, the IC_50_ for glyphosate is in the range of that for copper sulfate, significantly lower than the IC_50_ for fenhexamid and fenpropimorph, and ∼ 10‐fold higher than that for the fungicidal compound chlorothalonil.^16^, ^21^

### 3.3 Effects of seed treatments on AMF

One way of delivering active fungicidal or insecticidal substances directly to the crop plant and its surrounding area in the soil is by seed coating. Substances applied in this manner are active when AMF are still in the pre‐symbiotic phase and thus might severely impact the establishment of symbiosis. Studies on seed treatments identified factors such as the mode of action of the active substances, the host plant species and genotypes and the AMF communities as being relevant for the observed effects.^22^, ^23^ In corn, soybean and oat, mixtures of locally systemic (trifloxystrobin, pyraclostrobin, azoxystrobin, penflufen, sedaxane), xylem‐mobile systemic (mefenoxam, prothioconazole, metalaxyl, tebuconazole, triticonazole, prothioconazole) and contact active (fludioxonil) ingredients did not reduce AM root colonization and the P contents of plants compared with the control treatments.^22^ Seed treatment of peas with metalaxyl reduced root colonization irrespective of the inoculum type (native or commercial). In chickpea, a reduction in colonization was evident only for the commercial inoculum, indicating a different tolerance of native AMF species. Furthermore, there were no effects of captan on total AMF root colonization, but reduced Shannon diversity and species richness of AMF were identified, whereas thiram did not affect any of these parameters.^23^ Among the different responses of AMF species to pesticides, their individual preferences in host plant colonization^23^ might explain these results. Thus, studies on whole AMF communities are necessary to reveal such complex interactions and to identify the putative adaptability of AMF communities to pesticide use.

### 3.4 Direct and indirect effects on active AM symbiosis

Later in the season, after germination and successful establishment of the symbiosis, extraradical fungal units such as mycelia and spores can be exposed to active substances, which could reduce symbiotic activity and thus reduce P transport to the plant. Intraradical structures of AMF, such as hyphae, arbuscules and vesicles, can be exposed to active substances *via* root or hyphal uptake from the soil or when the substances are transported systemically from above‐ground plant parts to the roots. Furthermore, indirect effects on AMF can occur when the application of pesticides alters the metabolism of the host plant (Fig. 1). This alteration depends on the type of pesticide and varies among substances. Positive growth‐promoting effects triggered by changes in the hormonal balance (e.g., with strobilurines)^24^ can occur and might alter the interaction between AMF and the host plant. Although little is known about such indirect effects *via* changes in plant physiology, these changes might be key drivers for negative and positive effects on AM symbiosis. Furthermore, undesirable (e.g., due to intolerances of specific crop varieties) and intended phytotoxic effects of pesticides, for instance, might have indirect negative effects on AM symbiosis. Although phytotoxic effects should not occur under good agricultural practice, i.e., recommended dosage and proper application settings using fungicides or insecticides, they are of course a key factor in eliminating weeds.

The reported effects of pesticides on AM symbiosis are very diverse, and even when the same substance was investigated, the results are often contradictory (overview of studies in Table S1). There are many reasons for the documented differences in pesticides at similar concentrations among experiments: the field soils used, the tested crop plant, the cropping history, the AMF species, the test duration, the application mode, the applied volume and/or the final composition of the test substance.

For example, it was shown using *in vitro* systems that direct contact of the extraradical structures with fungicidal substances such as propiconazole (sterol biosynthesis inhibitor) was necessary to reduce hyphal length (threshold level of 0.02 mg L^−1^) and the spore number (threshold level of 0.2 mg L^−1^) in the hyphal compartment (Table S2).^25^ In pot experiments, the results are contradictory, ranging from neutral^26^ to negative effects on the activity of external hyphae^27^ and hyphal P uptake^28^ when propiconazole in its formulated product (Tilt 250EC®) was applied to the soil. Internal fungal structures were not affected in these studies. In detail, the volume of the applied fungicide solution differed between experiments. This difference might have caused a different spatial distribution within the substrate and thus different exposure situations. However, it should be noted that negative effects did not occur under the recommended field rates, and the dose is an important factor. Furthermore, it would be interesting to test the same parameters more than a few days after fungicide application, as the effects could be transient. In that way, we could learn more about the kinetics of those effects and better understand the field state.

Another pesticide example is fenpropimorph, which is also a sterol biosynthesis inhibitor. *In vitro* root colonization, spore production and extraradical hyphal length were negatively affected by fenpropimorph at a concentration of 0.02 mg L^−1^.^16^, ^29^ Furthermore, germ tube length, mycelial architecture, alkaline phosphatase activity and succinate dehydrogenase were inhibited in a dose‐dependent manner.^16^, ^30^ In pot experiments, negative effects on alkaline phosphatase activity were evident only at a concentration of 125 µg a.i. g^−1^ soil,^27^ which corresponds to application rates 100 times the recommended field rate. However, in another experiment, hyphal P uptake was not affected at any concentration.^28^ Again, factors such as soil, AMF community and applied fungicide solution volume might cause these differences. The examples of propiconazole and fenpropimorph show that *in vitro* tests can provide insight into the principal hazard potential of a substance, but nevertheless, realistic experiments based on the mode of application and field rates need to be performed to analyze the given risk. Furthermore, differences between the active substance and its corresponding formulation need to be discerned. When flutolanil (an inhibitor of fungal respiration) was tested as its formulated product (Monarch®), the threshold level for the reduction of root colonization was reduced to 0.1 mg L^−1^ (formulation) compared with flutolanil alone (1 mg L^−1^ a.i.).^8^ This result is not surprising because formulations can include compounds that enhance the biological activity of the active ingredient or have a biological activity of their own.^31^

## 4 EFFECTS ON AMF SPECIES DIVERSITY

A species‐specific reaction has already been documented for some herbicides, insecticides and fungicides. For instance, oxamyl, a systemic insecticide, did not affect a native *Glomus* sp. isolate, but root colonization was reduced for a commercial *Funneliformis mosseae* inoculum.^32^ Azadirachtin is a triterpenoid produced by species of the tree *Azadirachta* spp., and it functions as a feeding deterrent and growth disruptor for many insects. This product selectively inhibits the AM fungus *C. etunicatum* and causes a significant shift in the AMF community in the field.^33^ For the herbicide glyphosate, which has been shown to affect AMF at concentrations above the recommended application rate, the inhibitory effect on germination and hyphal growth was more pronounced for *C. etunicatum* than for *S. heterogama* and *G. margarita*.^19^ An AMF species‐specific response to glyphosate has also been determined in the field. In grasslands, a clear species‐specific effect was also shown. Whereas the spore viability of *F. mosseae* and *C. etunicatum* was similar in all treatments, the spore viability of *F. caledonium* and *F. constrictum* was reduced in glyphosate‐treated (field rate) plots.^34^ This result reflects the different sensibilities of certain AMF strains, which have also been shown for different soil tillage and management practices.^2^, ^3^

Limitations to studying AMF species diversity *via in vitro* experiments are the limited suitability of different AMF taxa for such experiments and the laborious establishment and time‐consuming maintenance of such cultures. *R. irregularis* has been most frequently studied with *in vitro* experiments (Table S2) but cannot reflect the behavior of other AMF species occurring in the field. However, the availability of AMF‐specific primer sets, together with different high‐throughput sequencing tools, allows for monitoring of the effects of pesticides on AMF communities in the field.^35^, ^36^, ^37^, ^38^, ^39^ Furthermore, the use of quantitative PCR allows quantitative monitoring of key taxa.^11^, ^40^ Based on these tools, there is evidence that AMF taxa react differently to certain pesticides in relation to their mode of action^23^ and concentration.^11^ For instance, metalaxyl‐based systemic fungicides exerted negative effects on *Septoglomus viscosum*, *Glomus hoi* and *Rhizophagus prolifer* in pea and on *Acaulospora* in chickpea.^23^ Other species such as *F. mosseae* and *C. claroideum* appeared to be more tolerant to metalaxyl‐based fungicides.^23^ In the case of captan‐based fungicides, the colonization of pea and chickpea with *Se. viscosum* and *Glomus* sp. was stimulated. Overall, these pesticide effects on AMF development can be linked to an existing specificity of fungicidal effects on AMF species. The underlying mechanisms of these differential fungicidal effects on different AMF species have not yet been elucidated.^23^

## 5 DOSES OF PLANT PROTECTION PRODUCTS

Good agricultural practices imply the application of all plant protection products at specific approved doses that have been reviewed and authorized by regulatory authorities. Under field conditions, both the dose of the formulated product in kg or L per volume water and the treated area must be adapted to the application technique for application of an efficient dose to the field. Major differences occur among application techniques for different crop plants (e.g., agricultural field crops, orchards and vineyards). A standard for dose expression of plant protection products has already been defined.^41^ However, comparison of the available studies is only possible to a limited degree. In particular, in pot experiments, neglect of the application area might lead to a potential overdosage compared with the recommended field rate. Furthermore, spatial expansion of the mycorrhizal network is very restricted in pot experiments but not under field conditions where the fungal entity can build an extensive network. For example, nicosulfuron has been tested as a potential indicator of pesticide microbial toxicity.^42^ In pot experiments at a concentration 10‐fold above the recommended field rate, this herbicide did not significantly affect AMF root colonization of maize plants; however, when applied at rates well above the recommended field rate (100‐fold, 1000‐fold), AMF hyphal colonization was significantly reduced. The result was inconsistent in an additional growth cycle of the experiment, and the colonization was even higher at a 1000‐fold rate; in a further three cycles, it was again reduced. By contrast, the accompanying field experiment with application of up to five‐fold the field recommended rate did not reduce the colonization rate of maize plants and did not alter the AMF community. There is evidence to suggest that the mycorrhizal network is able to compensate for possible negative effects, even at high doses, and is highly responsible for the resilience of AMF exposed to pesticides. Moreover, there are some indications that this resilience is not limited to AMF but can be conferred to the crop. Phenmedipham did not affect mycorrhizal colonization of lucerne, wheat and sorghum, and the beneficial effect of AMF also helped the crop plant recover from the deleterious effect of the herbicide.^43^

Another feature of dose dependency is the ability of a substance to react as an inhibitory and stimulatory compound. This concept is defined as ‘hormesis’.^44^ Depending on their mode of action, herbicides reduce weeds selectively while leaving the crop plant relatively unharmed, or kill all plants that come into direct contact with the active compound. Interestingly, at a sublethal dose, herbicides are not inhibitory but can stimulate other organisms. The hormetic effects of herbicides on plants are well documented.^44^, ^45^, ^46^ To date, little attention has been paid to hormesis in AMF–pesticide studies. However, reported dose‐dependent effects^18^, ^47^ may be attributed not only to hormesis, but also to an indirect effect *via* the plant. In‐depth studies are needed to elucidate this principle in AMF–plant–pesticide interactions.

## 6 STRATEGIES TO DEAL WITH PESTICIDES

As recently reviewed, AMF have evolved different strategies to deal with abiotic stresses such as organic pollutants and heavy metals.^48^ These strategies include morphological adaptation, protective molecules (antioxidants, chaperones, trehalose), pollutant transport, compartmentation, transformation and changes in gene expression. However, knowledge about the mechanisms of dealing with pesticides is limited, because the studies on AMF and pesticides are mostly descriptive rather than mechanistic.

Copper (Cu) is both a soil pollutant and used in plant protection products. Copper‐based fungicides have been widely used for more than a century and are included in crop protection strategies for integrated and organic agriculture. However, Cu accumulates in soil and may lead to toxic effects on plants and other soil biota when present at high concentrations. The effects of Cu on AMF have been mainly investigated with regard to metal toxicity, alleviation of plant stress occurring in highly contaminated soils and phytoremediation.^49^, ^50^ AMF have evolved different strategies, such as avoidance and compartmentation, to tolerate Cu‐contaminated environments.^51^ These strategies include the restriction of Cu to the cytoplasm, intracellular complexation in the cytosol and translocation to the vacuole. Furthermore, Cu can be translocated to specific fungal structures, such as extraradical spores and intraradical vesicles. Experiments with 150, 300 and 450 mg Cu kg^−1^ soil showed that the amount of blue‐colored spores (i.e., accumulation of Cu) of *C. claroideum* increased with Cu concentration. Spore vitality started decreasing at a concentration of 300 mg but did not decrease below 50% of the control treatment, even at a concentration of 450 mg Cu kg^−1^.^52^ This finding supports the assumption that this compartmentation strategy assures the survival of the fungal entity. In an *in vitro* ‘worst case’ scenario, *R. irregularis* associated with Ri T‐DNA‐transformed carrot roots was challenged with different concentrations of copper sulfate. Extraradical mycelial growth and extraradical sporulation were reduced in a dose‐dependent manner (IC_50_ 0.6 and 0.3 ppm, respectively).^21^ However, when Cu‐based products were applied to the soil, root colonization was not affected or was only moderately affected (Table S1).^53^, ^54^ Thus, the use of Cu‐based plant protection products appears to be compatible with AMF due to the strategies evolved by AMF for Cu‐contaminated environments. Nevertheless, AMF can reduce the negative effects exhibited by Cu toxicity on the host plant. However, the compatibility of AMF with Cu is dose‐dependent, and Cu accumulation in soils remains an environmental issue that needs to be addressed.

Further indications that AMF have evolved strategies that overcome adverse situations when exposed to pesticides are also observed for herbicides. It has been documented that some herbicide treatments affect AMF root colonization or spore number within only the first few days but will become balanced after a few weeks.^55^, ^56^ Thus, these studies lead to the conclusion that the harmful effect of these herbicides on weeds has an impact on the mycorrhizal network connecting the plant roots of weed and crop plants, and the crop plants seem to be able to compensate for this effect over a few weeks. However, these effects are highly dependent on the crop plant species. Whereas AMF in cowpea and common beans were able to recover, this was not the case for lupin.^55^ Moreover, such an effect was shown only for a conventional tillage system; under no till, AMF was not affected by the pesticides, and the AMF colonization was clearly higher under no till than under conventional tillage practices.^56^ In a greenhouse study, a glyphosate treatment and mechanical soil disturbance were compared regarding the AMF colonization of the subsequent crop. The former provides an intact and the latter a disrupted extraradical mycelium. The soil disturbance resulted in detrimental effects on AMF colonization.^57^ Thus, well‐established fungal networks can support the survival of AMF during pesticide exposure.

## 7 INTERACTIONS OF AMF WITH OTHER SOIL MICROORGANISMS

After successful establishment of the symbiosis with the host plants, AMF extend their extraradical hyphal network into the surrounding soil. This hyphal network absorbs nutrients from the surrounding soil, interconnects the roots of different plant individuals and shares the habitat with other soil microorganisms, such as P‐solubilizing bacteria.^58^ There is evidence that the microbial community of bulk soil differs from that in the hyphosphere (i.e., microenvironment influenced by hyphae themselves) and attached to the AM hyphal surface.^59^, ^60^, ^61^ Furthermore, AMF distribute recently assimilated C from the host plant to the soil microbial community *via* the hyphosphere.^62^ Whether AMF can actively shape their specific microorganism community by releasing certain key substances or whether this is due to common bacterial mycophagy on fungus‐derived substrates remains unknown.^63^, ^64^ Furthermore, there are implications that the microorganism community composition supports the survival of AMF under adverse abiotic conditions. For instance, the intimate relationship between AMF and certain bacterial species (e.g., *Pseudomonas* spp., *Bacillus* spp. and *Paenibacillus* spp.) has been documented in several cases, which also led to the introduction of the concept of ‘mycorrhiza helper bacteria’.^63^, ^65^ Apart from stimulating spore germination and mycelial growth, mycorrhiza helper bacteria reduce soil‐mediated stress, such as toxic concentrations of heavy metals.^65^ Soil‐living bacteria are important key players in pesticide biodegradation, and successful degraders accumulate upon repeated pesticide application.^66^ To date, it is not known whether successful biodegraders can also function as mycorrhiza helper bacteria. However, it is tempting to speculate that there is a connection and that AMF might even recruit biodegraders of pesticides. Conversely, pesticide degradation activity is not homogenously distributed at the field scale.^10^ In fact, there is high spatial variability. One aspect responsible for this spatial variability is the spatial distribution of bacteria. Apart from passive transport, bacteria move actively *via* flagella, depending on water films >1.5 µm.^67^ Fungal hyphae can provide such water films and can act as ‘fungal highways’ for the distribution of bacterial biodegraders within the soil.^68^, ^69^ In particular, in no‐till scenarios where fungal networks remain intact, these common mycorrhizal networks might play a crucial role in spreading pesticide‐degrading bacteria.

## 8 CONCLUDING REMARKS

Overall, based on the presented studies, the AMF response to pesticides is clearly substance‐ and dose‐dependent. Hence, the target site, the application mode in the field and the soil parameters determine the amount of substance/formulation reaching the AMF fungal structures and the principal risk of exposure. The mainly descriptive data have been generated in *in vitro*, greenhouse and field studies; however, little is known about AMF species diversity in colonized roots of crop plants, particularly under pesticide application. Molecular tools based on AMF‐specific primers and high‐throughput sequencing will allow us to gain more knowledge about the effects at the species level. Thus, the combination of (long‐term) field experiments with different tillage systems, standard IPM and molecular tools will provide more information on different species diversity parameters. Moreover, there is a strong need to study the effects of the combination of AMF‐friendly soil management and pesticide application on plant health and performance, which are the key considerations for pesticide application. This approach will be a further step towards responsible agricultural practices.

## Supporting information


**Appendix S1.** Supplemental references.

**Table S1.** Studies on AMF and pesticides.

**Table S2.** Different pesticide classes (F = fungicide, H = herbicide, I = insecticide) grouped according to their mode of action and their impact on AMF *in vitro*.
