# Supplementary material for: Arbuscular mycorrhizal fungi and their response to pesticides
Source: Pest Manag Sci. 2018 Oct 29;75(3):583–90. doi: 10.1002/ps.5220 (PMC6587947; doi:10.1002/ps.5220)
Supplement: Supplementary file 1 — Appendix S1. Supplemental references. [file PS-75-583-s003.pdf]

Supplemental References

70 Assaf TA, Turk MA and Hameed KM, Impact of olive pomace wastes and fungicide treatment on indigenous arbuscular mycorrhizal fungi associated with chickpea (*Cicer arietinum* L.) under field conditions. *Aust J Crop Sci* **3**:6-12 (2009).

71 Carrenho R, Trufem SFB and Bononi VLR, Arbuscular mycorrhizal fungi in *Citrus sinensis*/*C. limon* treated with fosetyl-Al and metalaxyl. *Mycol Res* **102**:677-682 (1998).

72 Afek U, Menge JA and Johnson ELV, Interactions among mycorrhizae, soil solarization, metalaxyl, and plants in the field. *Plant Dis* **75**:665-671 (1991).

73 Sukarno N, Smith SE and Scott ES, The effect of fungicides on vesicular–arbuscular mycorrhizal symbiosis: I. The effects on vesicular–arbuscular mycorrhizal fungi and plant growth. *New Phytol* **125**:139-147 (1993).

74 Fontanet X, Estaún V, Camprubí A and Calvet C, Fungicides added to potting substrate affect mycorrhizal symbiosis between a peach-almond rootstock and *Glomus* sp. *HortScience* **33**:1217-1219 (1998).

75 Jabaji-Hare SH and Kendrick WB, Response of an endomycorrhizal fungus in *Allium porrum* L. to different concentrations of the systemic fungicides, metalaxyl (Ridomil®) and fosetyl-al (Aliette®). *Soil Biol Biochem* **19**:95-99 (1987).

76 Shetty PK and Magu SP, Influence of metalaxyl on *Glomus fasciculatum* associated with wheat (*Triticum aestivum* L.). *Curr Sci* **72**:275-277 (1997).

77 Seymour NP, Thompson JP and Fiske ML, Phytotoxicity of fosetyl Al and phosphonic acid to maize during production of vesicular-arbuscular mycorrhizal inoculum. *Plant Dis* **78**:441-446 (1994).

78 Bary F, Gange AC, Crane M and Hagley KJ, Fungicide levels and arbuscular mycorrhizal fungi in golf putting greens. *J Appl Ecol* **42**:171-180 (2005).

79 Rhodes L and Larsen P, Effects of fungicides on mycorrhizal development of creeping bentgrass. *Plant Dis* **65**:145-147 (1981).

80 von Alten H, Lindemann A and Schönbeck F, Stimulation of vesicular-arbuscular mycorrhiza by fungicides or rhizosphere bacteria. *Mycorrhiza* **2**:167-173 (1993).

81 Cardenas-Flores A, Cranenbrouck S, Draye X, Guillet A, Govaerts B and Declerck S, The sterol biosynthesis inhibitor molecule fenhexamid impacts the vegetative compatibility of *Glomus clarum*. *Mycorrhiza* **21**:443-449 (2011).

82 Channabasava, Lakshman HC and Jorquera MA, Effect of fungicides on association of arbuscular mycorrhiza fungus *Rhizophagus fasciculatus* and growth of Proso millet (*Panicum miliaceum* L.). *J Soil Sci Plant Nutr* **15**:35-45 (2015).

83 Perrin R and Plenchette C, Effect of some fungicides applied as soil drenches on the mycorrhizal infectivity of two cultivated soils and their receptiveness to *Glomus intraradices*. *Crop Protect* **12**:127-133 (1993).

84 Kough JL, Gianinazzi-Pearson V and Gianinazzi S, Depressed metabolic activity of vesicular-arbuscular mycorrhizal fungi after fungicide applications. *New Phytol* **106**:707-715 (1987).

85 Schreiner RP and Bethlenfalvay GJ, Mycorrhizae, biocides, and biocontrol. 4. Response of a mixed culture of arbuscular mycorrhizal fungi and host plant to three fungicides. *Biol Fertility Soils* **23**:189-195 (1996).

86 Schreiner RP and Bethlenfalvay GJ, Plant and soil response to single and mixed species of arbuscular mycorrhizal fungi under fungicide stress. *Appl Soil Ecol* **7**:93-102 (1997).

87 Schreiner RP and Bethlenfalvay GJ, Mycorrhizae, biocides, and biocontrol 3. Effects of three different fungicides on developmental stages of three AM fungi. *Biol Fertility Soils* **24**:18-26 (1997).

88 Aziz T, Habte M and Yuen JE, Inhibition of mycorrhizal symbiosis in *Leucaena leucocephala* by chlorothalonil. *Plant Soil* **131**:47-52 (1991).

89 Habte M, Aziz T and Yuen JE, Residual toxicity of soil-applied chlorothalonil on mycorrhizal symbiosis in *Leucaena leucocephala*. *Plant Soil* **140**:263-268 (1992).

90 Sukarno N, Smith FA, Smith SE and Scott ES, The effect of fungicides on vesicular-arbuscular mycorrhizal symbiosis. *New Phytol* **132**:583-592 (1996).

91 Rejon A, Garcia-Romera I, Ocampo JA and Bethlenfalvay GJ, Mycorrhizal fungi influence competition in a wheat - Ryegrass association treated with the herbicide diclofop. *Appl Soil Ecol* **7**:51-57 (1997).

92 Mujica MT, Fracchia S, Ocampo JA and Godeas A, Influence of the herbicides chlorsulfuron and glyphosate on mycorrhizal soybean intercropped with the weeds *Brassica campestris* or *Sorghum halepensis*. *Symbiosis* **27**:73-81 (1999).

93 Zaller JG, Heigl F, Ruess L and Grabmaier A, Glyphosate herbicide affects belowground interactions between earthworms and symbiotic mycorrhizal fungi in a model ecosystem. *Scientific Reports* **4**2014).

94 de Freitas MAM, Silva DV, Guimarães FR, Leal PL, de Souza Moreira FM, da Silva AA, et al., Biological attributes of soil cultivated with corn intercropped with Urochloa brizantha in different plant arrangements with and without herbicide application. *Agric, Ecosyst Environ* **254**:35-40 (2018).

95 Makarian H, Poozesh V, Asghari HR and Nazari M, Interaction effects of arbuscular mycorrhiza fungi and soil applied herbicides on plant growth. *Commun Soil Sci Plant Anal* **47**:619-629 (2016).

96 Bethlenfalvay GJ, Mihara KL, Schreiner RP and McDaniel H, Mycorrhizae, biocides, and biocontrol. 1. Herbicide-mycorrhiza interactions in soybean and cocklebur treated with bentazon. *Appl Soil Ecol* **3**:197-204 (1996).

97 Bethlenfalvay GJ, Schreiner RP, Mihara KL and McDaniel H, Mycorrhizae, biocides, and biocontrol. 2. Mycorrhizal fungi enhance weed control and crop growth in a soybean-cocklebur association treated with the herbicide bentazon. *Appl Soil Ecol* **3**:205-214 (1996).

98 Baumgartner K, Fujiyoshi P, Smith R and Bettiga L, Weed flora and dormant-season cover crops have no effects on arbuscular mycorrhizae of grapevine. *Weed Res* **50**:456-466 (2010).

99 Baumgartner K, Smith RF and Bettiga L, Weed control and cover crop management affect mycorrhizal colonization of grapevine roots and arbuscular mycorrhizal fungal spore populations in a California vineyard. *Mycorrhiza* **15**:111-119 (2005).

100 Alguacil MM, Torrecillas E, García-Orenes F and Roldán A, Changes in the composition and diversity of AMF communities mediated by management practices in a Mediterranean soil are related with increases in soil biological activity. *Soil Biol Biochem* **76**:34-44 (2014).

101 Zhang Y, Wang L, Yuan Y, Xu J, Tu C, Fisk C, et al., Irrigation and weed control alter soil microbiology and nutrient availability in North Carolina Sandhill peach orchards. *Sci Total Environ* **615**:517-525 (2018).

102 Stoklosa A, Nandanavanam R, Puczel U and Upadhyaya MK, Influence of isoxaflutole on colonization of corn (*Zea mays* L.) roots with arbuscular mycorrhizal fungus *Glomus intraradices*. *Can J Plant Sci* **91**:143-145 (2011).

103 Pasaribu A, Mohamad RB, Hashim A, Rahman ZA, Omar D and Morshed MM, Effect of herbicide on sporulation and infectivity of vesicular arbuscular mycorrhizal (glomus mosseae) symbiosis with peanut plant. *J AnimPlant Sci* **23**:1671-1678 (2013).

104 Savin MC, Purcell LC, Daigh A and Manfredini A, Response of mycorrhizal infection to glyphosate applications and P fertilization in glyphosate-tolerant soybean, maize, and cotton. *J Plant Nutr* **32**:1702-1717 (2009).

105 Druille M, Cabello MN, Omacini M and Golluscio RA, Glyphosate reduces spore viability and root colonization of arbuscular mycorrhizal fungi. *Appl Soil Ecol* **64**:99-103 (2013).

106 Druille M, García-Parisi PA, Golluscio RA, Cavagnaro FP and Omacini M, Repeated annual glyphosate applications may impair beneficial soil microorganisms in temperate grassland. *Agric, Ecosyst Environ* **230**:184-190 (2016).

107 Druille M, Omacini M, Golluscio RA and Cabello MN, Arbuscular mycorrhizal fungi are directly and indirectly affected by glyphosate application. *Appl Soil Ecol* **72**:143-149 (2013).

108 Watrud LS, King G, Londo JP, Colasanti R, Smith BM, Waschmann RS, et al., Changes in constructed Brassica communities treated with glyphosate drift. *Ecol Appl* **21**:525-538 (2011).

109 Yang Y, Wang H, Tang J and Chen X, Effects of weed management practices on orchard soil biological and fertility properties in southeastern China. *Soil Tillage Res* **93**:179-185 (2007).

110 Beltrano J, Ruscitti M, Arango C and Ronco M, Changes in the accumulation of shikimic acid in mycorrhized *Capsicum annuum* L. grown with application of glyphosate and phosphorus. *Theor Exp Plant Physiol* **25**:125-136 (2013).

111 Ronco MG, Ruscitti MF, Arango MC and Beltrano J, Glyphosate and mycorrhization induce changes in plant growth and in root morphology and architecture in pepper plants (Capsicum annuum L.). *J Hortic Sci Biotechnol* **83**:497-505 (2008).

112 Nivelle E, Verzeaux J, Chabot A, Roger D, Chesnais Q, Ameline A, et al., Effects of glyphosate application and nitrogen fertilization on the soil and the consequences on aboveground and belowground interactions. *Geoderma* **311**:45-57 (2018).

113 Helander M, Saloniemi I, Omacini M, Druille M, Salminen J-P and Saikkonen K, Glyphosate decreases mycorrhizal colonization and affects plant-soil feedback. *Sci Total Environ* **642**:285-291 (2018).

114 Zaller JG, Cantelmo C, Santos GD, Muther S, Gruber E, Pallua P, et al., Herbicides in vineyards reduce grapevine root mycorrhization and alter soil microorganisms and the nutrient composition in grapevine roots, leaves, xylem sap and grape juice. *Environ Sci Pollut Res.* DOI: 10.1007/s11356-018-2422-3 (2018).

115 Garcia-Romera I and Ocampo JA, Effect of the herbicide MCPA on VA mycorrhizal infection and growth of *Pisum sativum*. *Z Pflanzenernähr Bodenkd* **151**:225-228 (1988).

116 Lutgen ER and Rillig MC, Influence of spotted knapweed (*Centaurea maculosa*) management treatments on arbuscular mycorrhizae and soil aggregation. *Weed Sci* **52**:172-177 (2004).

117 Corkidi L, Bohn J and Evans M, Effects of bifenthrin on mycorrhizal colonization and growth of corn. *HortTechnology* **19**:809-812 (2009).

118 Vijayalakshmi M and Rao AS, Effects of six insecticides and one fungicide on the development of VAM fungi in peanut (*Arachis hypogaea* L.). *Zentralbl Mikrobiol* **148**:60-65 (1993).

119 Wan MT and Rahe JE, Impact of azadirachtin on *Glomus intraradices* and vesicular-arbuscular myocorrhiza in root inducing transferred DNA transformed roots of *Daucus carota*. *Environ Toxicol Chem* **17**:2041-2050 (1998).
