# Supplementary material for: Arbuscular mycorrhizal fungi and their response to pesticides
Source: Pest Manag Sci. 2018 Oct 29;75(3):583–90. doi: 10.1002/ps.5220 (PMC6587947; doi:10.1002/ps.5220)
Supplement: Supplementary file 2 — Table S1. Studies on AMF and pesticides. [file PS-75-583-s002.pdf]

Supplemental Table 1: Studies on AMF and pesticides.

	**Substance** common name	**Applicat ion** 1=foliar 2=soil, 3=seed	**Code and Mode of action**		**Test systems**	**References**[§]
Fungicides	Metalaxyl	123	A1	Nucleic acid metabolism	pot, field	Cameron et al.[22], Jin et al.[23] Assaf et al.[70], Carrenho et al.[71], Afek et al.[72], Sukarno et al.[73], Fontanet et al.[74], Jabaji-Hare and Kendrick[75], Shetty and Magu[76], Hernandez-Dorrego and Pares[53], Seymour et al.[77]
	Pencycuron	3	B4	Cytoscleleton and motor protein	*in vitro*	Buysens et al.[8]
	Flutolanil	1 3	C2	Respiration	*in vitro*	Buysens et al.[8]
	Penflufen	3	C2	Respiration	pot	Cameron et al.[22]
	Sedaxane	3	C2	Respiration	pot	Cameron et al.[22]
	Azoxystrobin	1	C3	Respiration	*in vitro,* pot	Hernandez-Dorrego and Pares[53], Buysens et al.[8]
	Kresoxim-methyl	1	C3	Respiration	pot	Hernandez-Dorrego and Pares[53]
	Trifloxystrobin	1	C3	Respiration	pot	Cameron et al.[22]
	Pyraclostrobin	1	C3	Respiration	pot	Cameron et al.[22]
	Mepanipyrim	1	D1	Amino acid and protein synthesis	pot	Hernandez-Dorrego and Pares[53]
	Iprodione	1 3	E3	Signal transduction	pot, field	Hernandez-Dorrego and Pares[53], Sreenivasa and Bagyaraj[54], Bary et al.[78], Rhodes and Larsen[79]
	Etridiazole	2	F3	Lipid synthesis/transport; membrane	pot	Hernandez-Dorrego and Pares[53]
	Propamocarb	12	F4	Lipid synthesis/transport; membrane integrity	pot	Fontanet et al.[74], Hernandez-Dorrego and Pares[53]
	Propiconazole	1	G1	Sterol biosynthesis in membranes	*in vitro*, pot	Calonne et al.[25], Kjøller and Rosendahl[27], Kling and Jakobsen[26], Schweiger and Jakobsen[28], von Alten et al.[80]
	Prothioconazole	1 3	G1	Sterol biosynthesis in membranes	pot	Cameron et al.[22]
	Tebuconazole	1 3	G1	Sterol biosynthesis in membranes	pot	Cameron et al.[22]
	Triticonazole	1 3	G1	Sterol biosynthesis in membranes	pot	Cameron et al.[22]

	Fenpropimorph	1	G2	Sterol biosynthesis in membranes	*in vitro*, pot	Zocco et al.[16], Zocco et al.[30], Campagnac et al.[29], Kjøller and Rosendahl[27], Schweiger and Jakobsen[28]
	Fenhexamid	1	G3	Sterol biosynthesis in membranes	in vitro, pot	Zocco et al.[16], Zocco et al.[30], Campagnac et al.[29], Cardenas-Flores et al.[81], Hernandez-Dorrego and Pares[53]
	Copper oxychloride, copper sulfate	1	M1	Multi-site activity	in vitro, pot	Hernandez-Dorrego and Pares[53], Sreenivasa and Bagyaraj[54], Channabasava et al.[82], Wan et al.[21]
	Mancozeb	1	M3	Multi-site activity	pot	Hernandez-Dorrego and Pares[53], Sreenivasa and Bagyaraj[54], Channabasava et al.[82]
	Thiram	12	M3	Multi-site activity	pot	Jin et al.[23] ,Perrin and Plenchette[83], Sreenivasa and Bagyaraj[54]
	Captan	123	M4	Multi-site activity	pot	Jin et al.[23], Kough et al.[84], Schreiner and Bethlenfalvay[85-87], Sreenivasa and Bagyaraj[54], Channabasava et al.[82]
	Chlorothalonil	1	M5	Multi-site activity	pot	Hernandez-Dorrego and Pares[53], Aziz et al.[88], Habte et al.[89]
	Fosetyl-AI	12	P	Host plant defense induction	pot, field	Jabaji-Hare and Kendrick[75] Carrenho et al.[71], Hernandez-Dorrego and Pares[53], Sukarno et al.[73], Sukarno et al.[90]
Herbicides	Diclofop	1	A	Inhibition of acetyl CoA carboxylase	pot	Rejon et al.[91]
	Fluazifop	1	A	Inhibition of acetyl CoA carboxylase	field	Santos et al.[56]
	Chlorsulfuron	12	B	Inhibition of acetoacetate synthase	pot	Mujica et al.[92]
	Flazasulfuron	12	B	Inhibition of acetoacetate synthase	field	Zaller et al.[93]
	Nicosulfuron	12	B	Inhibition of acetoacetate synthase	field	Mujica et al.[92], de Freitas et al.[94]
	Metribuzin	12	C1	Inhibition of photosynthesis	pot	Makarian et al.[95]
	Phenmedipham	1	C1	Inhibition of photosynthesis	pot	Ocampo and Barea[43]
	Chlorotolouron	1	C2	Inhibition of photosynthesis	*in vitro*, pot	Dodd and Jeffries[18]
	Bentazon	1	C3	Inhibition of photosynthesis photosyst. II II	pot	Bethlenfalvay et al.[96,97]

	Bromoxynil	1	C3	Inhibition of photosynthesis	pot	Abd-Alla et al.[55]
	Ioxynil	2	C3	Indole acetic acid like action	*in vitro*, pot	Dodd and Jeffries[18]
	Fomesafen	12	E	Inhibition of protoporphyrinogen	field	Santos et al.[56]
	Oxyfluorfen	12	E	Inhibition of protoporphyrinogen oxidase	field	Baumgartner et al.[98,99], Alguacil et al.[100]
	Bifenox	12	E	Inhibition of protoporphyrinogen	*in vitro*, pot	Dodd and Jeffries[18]
	Flumioxazin	2	E	Inhibition of protoporphyrinogen	field	Zhang et al. 2018[101]
	Isoxaflutole	2	F2	Bleaching: Inhibition of 4-HPPD	pot	Stoklosa et al.[102]
	Mesotrione	12	F2	Bleaching: Inhibition of 4-HPPD	pot	Silveira et al.[47]
	Glyphosate	1	G	Inhibition of EPSP-synthase	*in vitro*, pot, field	Wan et al.[21], dos Santo Malty et al.[19]·Pasaribu et al.[20,103] Brito et al.[57], Mujica et al.[92], Savin et al.[104], Zaller et al.[93], Druille et al.[34,105-107] Watrud et al.[108], Yang et al.[109], Baumgartner et al.[98,99], Beltrano et al.[110], Ronco et al.[111], Nivelle et al.[112], Helander et al.[113], Zaller et al.[114]
	Glyfosinate	1	H	Inhibition of glutamine synthetase	field	Zaller et al.[114]
	MCPA	1	O	Indole acetic acid like action	pot	Garcia-Romera and Ocampo[115]
	Picloram	12	O	Indole acetic acid like action	*in vitro*, pot, field	Lutgen and Rillig[116], Lekberg et al.[17]
	Mecoprop	2	O	Indole acetic acid like action	*in vitro*, pot	Dodd and Jeffries[18]
	Clopyralid	2	O	Indole acetic acid like action	*in vitro*, pot	Lutgen and Rillig[116]
	2,4-D	2	O	Indole acetic acid like action	field	Lutgen and Rillig[116]
	Pelargonic acid	1	Z	unknown	pot	Zaller et al.[93]
Insecticides	Oxamyl	2	1A	Acetylcholinesteraseinhibitor	pot	Marin et al.[32]
	Bifenthrin	1	3A	Sodium channel modulator	pot	Corkidi et al.[117]
	Deltamethrin	1	3A	Sodium channel modulator	pot	Rivera-Becerril et al.[11]
	Cypermethrin	1	3A	Sodium channel modulator	pot	Vijayalakshmi and Rao[118]
	Pyrethrum	1	3A	Sodium channel modulator	pot, field	Ipsilantis et al.[33]
	Dimethoate	1	1B	Acetylcholinesteraseinhibitor	pot	Vijayalakshmi and Rao[118], Schweiger and Jakobsen[28]
	Spinosad	1	5	Nicotinic acetycholine receptor	pot, field	Ipsilantis et al.[33]
	Azadirachtin formulation)	1	UN	unknown	*in vitro*, pot, field	Wan and Rahe[119], Ipsilantis et al.[33]

[§]References not mentioned in the main text are available in the supplemental part.
