# Supplementary material for: Arbuscular mycorrhizal fungi and their response to pesticides
Source: Pest Manag Sci. 2018 Oct 29;75(3):583–90. doi: 10.1002/ps.5220 (PMC6587947; doi:10.1002/ps.5220)
Supplement: Supplementary file 3 — Table S2. Different pesticide classes (F = fungicide, H = herbicide, I = insecticide) grouped according to their mode of action and their impact on AMF in vitro. [file PS-75-583-s001.pdf]

Supplemental Table 2 Different pesticide classes (F=fungicide, H=herbicide, I=insecticide) grouped according to their mode of action and their impact on AMF *in vitro*.

**Substance common name**	**Target site and code)**	**Concentration (recommended application rate = rar)**	**Treat-ment**	**AMF species**	**Host plant**	**Effect on AMF negative (-), neutral (o), positive (+)**			**Type of AMF effects**	**References**
Pencycuron	F; B4	0.01, 0.5, 5 mg L^{-1} a.i.	medium	*Rhizophagus intraradices*	*Solanum tuberosum*	-	o		dose-dependent reduction of spore germination (never complete inhibition, fungistatic effect), reduced root colonization (threshold level 0.5 mg L^{-1})	Buysens et al.[8]
Flutolanil	F; C2	0.1, 1, 10 mg L^{-1} a.i.	medium	*Rhizophagus intraradices*	*Solanum tuberosum*	-	o		dose-dependent reduction of spore germination (never complete inhibition, fungistatic effect), reduced root colonization (threshold level 1 mg L^{-1}), impact on intraradical phase (systemic), but no effect at IC_{50} level of *R. solani*	Buysens et al.[8]
Azoxystrobin	F; C3	0.1, 1, 10, 100 mg L^{-1} a.i.	medium	*Rhizophagus intraradices*	*Solanum tuberosum*	-	o		dose-dependent reduction of spore germination (never complete inhibition, fungistatic effect), reduced root colonization (1 mg L^{-1})	Buysens et al.[8]

Propiconazole	F; G1	0.02-2 mg L^{-1}	medium	*Rhizophagus irregularis*	*Cichorium intybus (*Ri T-DNA-transformed roots*)*	-	o		reduction in hyphal length (threshold level 0.02 mg L^{-1}) and spore number (threshold level 0.2 mg L^{-1}), inhibition of 14alpha-demethylase, in HC when propiconazole was present, contact is necessary, effect on sterol composition of roots	Calonne et al.[25]
Fenpropimorph	F; G2	0.02, 0.2, 2, 20, 200 mg L^{-1}	medium	*Rhizophagus irregularis*	*Daucus carota (*Ri T-DNA-transformed roots*), Medicago trunculata*	-	o		neutral to negative effect on total root colonization (threshold level 0.02 mg L^{-1}), number of spores negative (threshold level 0.02 mg L^{-1}), fungistatic effect spore germination (IC_{50} 6.1 mg L^{-1}), germ tube length neutral to negative (dose-dependent, IC_{50} 6.4 mg L^{-1}), extraradical hyphal length reduced (threshold level 0.02 mg L^{-1}), at high concentration (20 mg L^{-1}) effects on mycelium architecture, ALP reduced (threshold level 0.2 mg L^{-1}), SDH reduced (threshold level 2 mg L^{-1},)	Zocco et al.[16], Zocco et al.[30], Campagnac et al.[29]

Fenhexamid	F; G3	0.02-200 mg L^{-1}	medium	*Rhizophagus intraradices*	*Daucus carota* (Ri T-DNA-transformed roots), *Medicago trunculata*	-	o		total root colonization not affected, number of spores neutral to negative (20 mg L^{-1}), fungistatic effect spore germination (IC_{50} 5.6 mg L^{-1}), germ tube length neutral to negative (dose-dependent, IC_{50} 9.3 mg L^{-1}), extraradical hyphal length not affected, arbuscules reduced (0.02 mg L^{-1}), ALP, SDH reduced (0.2 mg L^{-1})	Zocco et al.[16], Zocco et al.[30], Campagnac et al.[29]
Fenhexamid	F; G3	5, 10 mg L^{-1}	medium	*Rhizophagus clarus*	*Daucus carota* (Ri T-DNA-transformed roots)	-	o		continuous exposure over 5 generations, no effect on spore production but anastomosis was decreased (10 mg L^{-1})	Cardenas-Flores et al.[81]
Coppersulfate	F; M1	0.125, 0.625, 1.25, 6.25, 12.5 ppm	medium	*Rhizophagus intraradices*	*Daucus carota* (Ri T-DNA-transformed roots)	-	o		Extraradical mycelial growth (IC_{50} 0.6 ppm), extraradical mycelial sporulation (IC_{50} 0.3 ppm), reduction of root colonization at 6.25 ppm	Wan et al.[21]
Chloro-tolouron	H; C2	50 and 100 % rar; 1.75 & 3.5 µg mL^{-1}	inoculated pads; spraying (post sowing)	*Funneliformis geosporum, Glomus monosporum, Funneliformis mosseae*	*Triticum vulgare*		o	+	no effect on spore germination on incubation pads plates with herbicides in 50 and 100 % dose; no negative effect on AM infection of wheat at harvest; lower foliage and ear yields due to phytotoxicity	Dodd and Jeffries[18]
Glyphosate	H; G	0.125; 0.25; 0.5; 1.25 ppm	addition to culture media	*Rhizophagus intraradices*	*Daucus carota* (Ri T-DNA transformed roots)				determination of IC_{50} (EMG response indicator) 0.5 +/- 0.3; reduction of colonization at 0.125 ppm	Wan et al.[21]
Glyphosate	H; G	0-454 µM in culture media; equiv. 1.25-10 L ha^{-1}	addition to culture media;	*Gigaspora margarita, Claroideoglomus*	*Glycine max*		o		in vitro inhibition in culture media only when conc. greater than field dose; strain specific response; soil application up to	dos Santo Malty et al.[19]

			soil (pre sowing)	*etunicatum, Scutellospora heterogama*					highest conc. with no effect on AMF in soybean	
Bifenox & mecoprop	H; E; O	50 and 100 % rar; 1.0 & 2.0 $\mu g\ mL^{-1}$	inoculated pads; spraying (post sowing)	*Funneliformis geosporum, Glomus monosporum, Funneliformis mosseae*	*Triticum vulgare*		o	+	higher spore germination on incubation pads plates with herbicides in 100 % dose; no negative effect on AM infection of wheat at harvest; increased ear yield	Dodd and Jeffries[18]
Mecoprop, ioxynil, clopyralid	H; O; C3, O	50 and 100 % recommended application rate; 1.25 & 2.5 $\mu g\ mL^{-1}$ in control solution	inoculated pads; spraying (post sowing)	*Funneliformis geosporum, Glomus monosporum, Funneliformis mosseae*	*Triticum vulgare*		o	+	higher spore germination on incubation pads plates with herbicides in 100 % dose; no negative effect on AM infection of wheat at harvest; increased ear yield	Dodd and Jeffries[18]
Azadirachtin (pure, neem extracts and formulation)	I; UN	0.5-50 000 ppm depending on solvent and approach	added to minimal medium in petri dishes	*Rhizophagus intraradices*	*Daucus carota* (Ri T-DNA-transformed roots)		o		IC_{50} in minimal medium between 1 and >300 ppm depending on the solvent/formulation; significant effects on root colonization between 1.25 and 625 ppm depending on the solvent	Wan and Rahe[119]
